# The empirical estimate of the survival and variance using a weighted composite endpoint

**DOI:** 10.1186/s12874-023-01857-0

**Published:** 2023-02-06

**Authors:** Majid Nabipoor, Cynthia M. Westerhout, Sarah Rathwell, Jeffrey A. Bakal

**Affiliations:** 1 Provincial Research Data Services, Alberta Health Services, Edmonton, Alberta Canada; 2 Canadian VIGOUR Centre, University of Alberta, Alberta, Canada

**Keywords:** Survival Analysis, Time to Event, Reliability, Clinical Trials, Medical Statistics

## Abstract

**Background:**

Composite endpoints for estimating treatment efficacy are routinely used in several therapeutic areas and have become complex in the number and types of component outcomes included. It is assumed that its components are of similar asperity and chronology between both treatment arms as well as uniform in magnitude of the treatment effect. However, these assumptions are rarely satisfied. Understanding this heterogeneity is important in developing a meaningful assessment of the treatment effect.

**Methods:**

We developed the Weighted Composite Endpoint (WCE) method which uses weights derived from stakeholder values for each event type in the composite endpoint. The derivation for the product limit estimator and the variance of the estimate are presented. The method was then tested using data simulated from parameters based on a large cardiovascular trial. Variances from the estimated and traditional approach are compared through increasing sample size.

**Results:**

The WCE method used all of the events through follow-up and generated a multiple recurrent event survival. The treatment effect was measured as the difference in mean survivals between two treatment arms and corresponding 95% confidence interval, providing a less conservative estimate of survival and variance, giving a higher survival with a narrower confidence interval compared to the traditional time-to-first-event analysis.

**Conclusions:**

The WCE method embraces the clinical texture of events types by incorporating stakeholder values as well as all events during follow-up. While the effective number of events is lower in the WCE analysis, the reduction in variance enhances the ability to detect a treatment effect in clinical trials.

**Supplementary Information:**

The online version contains supplementary material available at 10.1186/s12874-023-01857-0.

## Introduction

Shrinking mortality rates and ever-tightening economic constrains have forced many randomized clinical trials (RCTs) to adopt the use of composite endpoints as a means of easily incorporating multiple clinically meaningful component endpoints and increasing the overall event rate and thus the power of the analysis. In cardiovascular clinical trails, the endpoint may appear as a composite of multiple types of Major Adverse Cardiac Events (MACE) such as all-cause death, myocardial infarction, heart failure, and revascularization. The use of a composite endpoint allows the study to both have and increased overall event rate and permit the use of a single test to evaluate the effect of a study treatment, ultimately achieving reasonable and acceptable power. In its most common form, the composite endpoint is structured either as a proportion (i.e., binary with “1’ indicating achievement of any one of these events during follow-up) or as time to the first event.

While straightforward as a measurement, the composite endpoint may be susceptible to heterogeneity, which is not assumed in the design or traditional analysis of the trial. The types of heterogeneity which may be observed include several types: (1) Qualitative heterogeneity which relates to the direction and magnitude of the differences between the two groups (e.g., study therapy; which decreases the rate of death, but increases the likelihood of heart failure) [1] (2) Asperity heterogeneity, which pertains to the clinical relevance of the outcome, e.g., a subject with a myocardial infarction (MI) during followup or a subject who dies before the end of the study; and (3) Chronological heterogeneity in which the events and treatment effect may be different by event (e.g. cardiogenic shock may occur within hours, while other events, like heart failure, may take several days or weeks to manifest, reducing its likelihood of being the first event) [2].

The traditional approach, which uses time-to-first event, is simple in computation as it reduces to a survival analysis. However, by selecting the first event only it fails to address both the chronological and the asperity heterogeneity, as the events used are typically over-represented by the clinically minor event types (e.g., recurrent ischemia).

There have been several approaches designed to deconstruct the composite endpoints with varying degrees of success in addressing the specified sources of heterogeneity [3]. While methods such as those by Andersen and Gill [4] do capture all of the events, it was designed for events of similar asperity. In cardiovascular clinical trials this is rarely the experience. Consider a scenario where a subject experiences two non-fatal events and would thus be represented with two “like” events while a second subject first has a non-fatal event and then dies. These two subjects would be considered as being identical in the Andersen Gill Method. Another emerging method, the win-ratio (adapted from log rank testing by Pocock et al. [5] addresses asperity heterogeneity by assigning a hierarchy of ‘worst-events’, it does so only by counting only the first-worst event, thereby failing to capture other important clinical information, including prior events or multiple events of the same asperity [6].

The solution presented here is the Weighted Composite Endpoint (WCE) based on standard survival methodology incorporates weights per event type derived from clinical relevance (i.e., physician/trialist perspective) [7]. With this approach each non-fatal outcome counts only as a portion of the subject’s weight, allowing for the inclusion of multiple events, at varying times and help mitigate the differences in magnitude of effect among different treatment strategies.

This manuscript builds upon our previous work and addresses concerns raised by others [8]. The remainder of this manuscript is divided into 4 sections: In the Motivation section we provide a description of a problem common in many clinical trials; in the Weighted Composite Endpoint (WCE) section we provide the derivation of the product limit estimate and variance for using the weighted composite endpoint method. In the Case study: the ASSENT-3 Trial section we provide a simulation algorithm for generating multiple recurrent event data, and a case study based on a simulated data with parameters of a clinical trial using the *wcep* R package which is available for download on the Comprehensive R Archive Network (CRAN) at https://CRAN.R-project.org/package=wcep. Finally, the Discussion studies the effect of the weighting by WCE on the variance compared to traditional approach, and discusses the results and application of the weighted composite endpoint method [7, 9].

## Motivation

Large-Scale Multicenter Randomized Clinical Trials (RCT) are considered the gold standard of treatment evaluation of a new treatment. This methodology has been evolving particularly in the cardiac sciences. A critical part of this robust methodology is dependent on the choice of outcome to be evaluated. In the 1970s the mortality in the first 7 days following a heart attack was approximately 30%, through changes in quality of care and treatments to improve the return of blood flow to the heart the current mortality rate is typically 1-3% at 30 days. This change has allowed researchers to include non-fatal events in the outcome which allows the inclusion of other clinically relevant events and also the reduction of sample size to sufficiently power a trial.

Traditional time-to-event analysis assigns equal and full weight to the first-event regardless of type in the composite endpoint. This is counter-intuitive to many stakeholders . Prior studies in cardiology have shown that both subjects and providers agree that the asperity of shock is less than the asperity of death, and heart failure and REMI are both less severe than shock [7, 10]. The WCE method addresses this issue, by using weights on the outcomes to provide an empirical estimate.

For the purposes of this paper, we consider a hypothetical RCT in ST-segment elevation myocardial infarction (heart attack) subjects based on the ASSENT-3 Trial [11]. In this trial, subjects were randomly assigned into either the treatment or control arm. The primary outcome of the trial is a four-component MACE endpoint which is the composite of Recurrent Myocardial Infarction (REMI; second heart attack), Congestive Heart Failure (CHF), cardiogenic shock (SHK), or all-cause death (DTH). While many other definitions of MACE are possible, this one is sufficiently generic to demonstrate the challenges and various forms of heterogeneity faced in these types of RCT. In this trial, subjects are randomized into one of the treatment arms at the time of heart attack, then followed for a specified time (e.g., 30 days post MI) to examine rates of MACE events. The short-term follow up and nature of the disease stated in this trial allows us to ignore right censoring. Generally this type of trial would be analyzed using a time-to-first event or ‘event-free’ survival. We will use this scenario to examine the weighted composite endpoint as a means of incorporating the heterogeneity in the event types to make better use of the collected data.

The weights or stakeholder values in Table 1 are constructed based on a questionnaire of a Delphi panel of experts in cardiovascular medicine and experienced clinician-investigators, familiar with the development and interpretation of randomized clinical trials of subjects with acute coronary syndrome to evaluate the severity of a series of conventional clinical efficacy endpoints. The stakeholder values derived carefully, evaluated by sensitivity analysis, and compared with ASSENT-3 trial results [7]. The time to event and event rates listed in Table 1 reflects the three forms of heterogeneity in the data. In this table, We can see typical variability in event rates for this type of composite outcome. Due to the different types of event, it is difficult to expect that a new intervention will change the event rates uniformly. Additionally, the event timing may differ between events and intervention arms. Finally, the outcomes show the differences in the asperity, along with the prescribed weights.

## Weighted Composite Endpoint (WCE)

The WCE method is a weighted product limit estimate derived from the Kaplan-Meier or product-limit estimator $\hat{S}(t)$ for survival function *S*(*t*)

$$\begin{aligned} \hat{S}(t)=\prod\limits_{ t_j \leq t}\left( 1-\frac{d_j}{n_j}\right) , \end{aligned}$$

where $n_j$ is size of the risk set at the distinct time $t_j$ and $d_j$ is the number of death in the risk set at time $t_j$. In the standard Kaplan-Meier approach, the hazard rate $\frac{d_j}{n_j}$ is calculated based on the risk set or the uncensored subjects at time $t_j$. WCE modifies the product-limit estimator by assigning a weight to each type of event, which reflects severity of the event [12]. Incorporating these weights allows us to estimate survival probability for each subject at distinct time *t*, and we will show that the survival function becomes the mean of the individual survival probabilities provided in Table B2 of the Appendix. Using a weighted event means that a subject would have a ‘partial’ weight remaining following a non-fatal event, allowing both continued followup and the incorporation of multiple events per subject.

To calculate the weighted survival estimate, we index individual subjects by *i* for $i=1, \ldots , n$; where *n* is the sample size or the total number of subjects at the start time $t_0$. Let $t_0=0< t_1< \ldots<t_j<\ldots <t_J$ be the finite set of distinct observed event times; and suppose that *K* possible events, indexed by *k*, can occur for each subject at the distinct times $t_j$. In our example we consider, REMI, CHF, SHK as potentially recurrent events, and DTH as a terminal event. Therefore, $K=4$ possible events exist with the specific weight vector $W=\left( \textrm{w}_1, \ldots , \textrm{w}_k, \ldots , \textrm{w}_K \right) ^T$. The weight vector *W* reflects the asperity of *K* possible events, and $\textrm{w}$ is the component of vector *W*. $E_{ijk}$ denotes the event type *k*, at distinct time $t_j$ for subject *i*, and note that $E_{ij}=\left( E_{ij1}, \ldots , E_{ijk}, \ldots , E_{ijK}\right) ^T$.

To incorporate multiple events per subject, we consider them sequentially multiplicative. For instance, if subject *i* had a SHK event at time $t_1$ and a CHF event at time $t_2$, then the weighted survival probability at time $t_1$ is $(1-\textrm{w}_{SHK})$ and at time $t_2$ is $(1-\textrm{w}_{SHK})(1-\textrm{w}_{CHF})$. The estimate of the weighted survival function for subject *i* at time *t* becomes

1 $$\begin{aligned} \hat{S}_i(t)=\prod\limits_{j:\ t_j \le t}\left( 1-\textrm{w}_{ij}\right) , \end{aligned}$$

where $\textrm{w}_{ij}$ denotes the weight of event that happened for subject *i* at time $t_j$. These events happen as multiple consecutive events which are assumed to be independent. The multiplicative adjustment does not allow a single subject to accumulate more than 1.0 total events. Multiple events at the same time can also be considered this way. Note that with this method, the traditional analysis (i.e., time-to-first event) becomes a special case of the WCE where the weights are set equal to 1.

Through a simple example, (Table B1 Appendix) we can see that weighted composite endpoint [12] simplifies to an estimate in the form of a weighted average. This designed randomized controlled trial reports the events of REMI, CHF, SHK and DTH of 25 subjects followed for 30 days. The survival probabilities can be calculated based on Bakal’s heuristic method for each subject, (Table B2 Appendix); and we suggest, the interested reader has a careful attention to this table before continuing the notation. In this heuristic method: $d_j$ is sum of the differences of survival probabilities of current and previous time for all subjects, $d_j=\sum _{i=1}^n \left[ \hat{S}_i(t_{j-1})-\hat{S}_i(t_{j})\right]$; $n_j=\sum _{i=1}^n\hat{S}_i(t_{j-1})$, Table B2 in the  Appendix is analogous to the Kaplan-Meier estimate as the number of alive subjects up to time $t_j$; and the weighted survival estimate $\hat{S}(t_{j-1}) (1-d_j/n_j)$ is equal to $\frac{1}{n}\sum _{i=1}^n\hat{S}_i(t_j)$, (Table B2 Appendix). In fact

$$\begin{aligned} \hat{S}(t_{1})=\hat{S}(t_0) \cdot \left( 1-\frac{\sum _{i=1}^n \left[ \hat{S}_i(t_0)-\hat{S}_i(t_1)\right] }{\sum _{i=1}^n\hat{S}_i(t_0)} \right) =1 \cdot \left( 1-\frac{\sum _{i=1}^n \left[ 1-\hat{S}_i(t_1)\right] }{n} \right) =\frac{1}{n}\sum\limits_{i=1}^n\hat{S}_i(t_1); \end{aligned}$$

and by induction for $2, \dots ,j$, $\hat{S}(t_j)=\frac{1}{n}\sum _{i=1}^n\hat{S}_i(t_j)$. Note that all subjects in this example have full 30 days of followup except for those who died. This study focused on randomized clinical trials, and ignores censoring. By this representation, WCE works well as there are generally no right censored records in RCTs. Hence, we define the survival function at time *t* as the average of survival probabilities of all subjects at time *t*:

2 $$\begin{aligned} \hat{S}(t)=\frac{1}{n}\sum\limits_{i=1}^n\hat{S}_i(t)=\frac{1}{n}\sum\limits_{i=1}^n\prod\limits_{j: \ t_j \leq t}\left( 1-\omega _{ij}\right) , \end{aligned}$$

where $\omega _{ij}$ is a real value between zero and one, as the weight of the event occurred for subject *i* at time $t_j$. There are *K* possible events $E_{ij}=\left( E_{ij1}, \ldots , E_{ijk}, \ldots , E_{ijK}\right) ^T$ at time $t_j$ for subject *i*; and we define

3 $$\begin{aligned} \omega _{ij}:=W^TE_{ij}=\sum\limits_{k=1}^K{\textrm{w}_k E_{ijk}}, \end{aligned}$$

where $E_{ijk}=1$ if event *k* occurred at time $t_j$ for subject *i*, otherwise 0. The variable of $E_{ijk}$ has the following distribution

$$\begin{aligned} p_1^{E_{ij1}}p_2^{E_{ij2}} \ldots p_K^{E_{ijK}} \left( 1- \sum\limits_{k=1}^Kp_k \right) ^{1- \sum _{k=1}^K E_{ijk}}, \end{aligned}$$

which is a multinomial distribution with $n=1$. Note that $1- \sum _{k=1}^Kp_k$ is the probability of no event. Therefore

4 $$\begin{aligned} E\left[ \omega _{ij} \right] = W^TE\left[ E_{ij}\right] =W^T P\left( E_{ij} \right) := W^TP_j; \end{aligned}$$

where $$P\left( E_{ij} \right) =P_j$$ and

5 $$\begin{aligned} Var\left( \omega _{ij} \right) =W^TVar\left( E_{ij} \right) W =W^T \left[ diag(P_j)-P_jP_j^T \right] W, \end{aligned}$$

where $P_j$ is the vector probabilities of *K* types of events that can happen for subject *i* at time $t_j$. We use the maximum likelihood estimate for $P_j$. The estimate of $P_j$ at time $t_j$ is

6 $$\begin{aligned} \hat{P}_j=\frac{E_{.j}}{n_j}, \end{aligned}$$

where $E_{.j}$ is the vector of the number of *K* types of events that occurred in the time $t_j$; and $n_j$ is the size of risk set at time $t_j$. Note that $\frac{E_{.j}}{n_j}$ in Eq. (6) used for variance calculation is analogous to the Kaplan-Meier hazard rate $\frac{d_j}{n_j}$. Now we have developed the components required to calculate the variance of the weighted survival function $Var\left( \hat{S}(t)\right)$.

The variance of survival function $Var\left( \hat{S}(t)\right)$ is required to derive an approximate 95% confidence interval for the survival function *S*(*t*). The variance of $\hat{S}(t)$ based on Eq. (2) is

$$\begin{aligned} \frac{1}{n^2}\sum\limits_{i=1}^n{Var\left( \hat{S}_i(t) \right) }, \end{aligned}$$

since subjects are independent, and

$$\begin{aligned} Var\left( \hat{S}_i(t) \right) =Var\left( \prod\limits_{j: t_j \le t}\left( 1-\omega _{ij}\right) \right) , \end{aligned}$$

which leads to (proof in the Appendix)

$$\begin{aligned} Var\left( \hat{S}(t) \right) \approx \frac{1}{n^2} \sum\limits_{i=1}^n\hat{S}_i^2(t)\sum\limits_{j: t_j \leq t} \frac{1}{(1-W^TP_j)^2}W^T \left[ diag(P_j)-P_jP_j^T \right] W. \end{aligned}$$

Given that the survival function $\hat{S}(t)$ defined as an average in (2), normality applies for large *n*, and an approximate C.I. with confidence level $(1-\alpha )$ is $\hat{S}(t) \pm z_{\frac{\alpha }{2}}sd\left( \hat{S}(t) \right)$.

## Case study: the ASSENT-3 Trial

As a case study in the application of the Weighted Composite Endpoint Method we developed a simulation based the events in a previously conducted trial in ST-Segment elevation myocardial infarction. The original ASSENT-3 [13] database housed at the Canadian VIGOUR Centre in Edmonton Alberta and was made available in de-identified form through request to the organization. The ASSENT-3 Trial enrolled 6095 subjects and randomized them 1:1:1 into three treatment groups. For the purposes of the case study, we consider two of the groups and base the estimates of event rates and timing ton the results of the  1500 subjects in receiving each type of therapy [13]. These data have been used previously in direct analysis and simulation studies [7, 12, 14]. The event rates of treatment and control groups, and mean time-to-event are given in Table 1. The event rates are based on ASSENT-3 randomized controlled trial and assumed to be independent. Hence, multiple events and recurrent events occur independently for simulated experiment data.

**Table 1:** Event rates and time-to-events are ASSENT-3’s results, and weights for weighted composite endpoints are given by Bakal et al. [12]

Outcome	Mean Time to Event	Event Rates (Qualitative)		Weight	Probable Outcome
	(Chronology)	Treatment	Control		(Asperity)
REMI	3.0 days	6%	3%	0.2	Longer rehabilitation
CHF	2.4 days	5%	4%	0.3	Chronic Condition, Shortness of breath, fatigue
Shock	2.3 days	4%	5%	0.5	Organ damage, device implantation
Death	5.5 days	3%	6%	1.0	Death

Simulation for multiple recurrent events is challenging. This simulation leverages the R packages *survsim* of Morina et al. [15] and *simsurv* of Brilleman et al. [16]. These packages are able to generate data for recurrent events or multiple events. However, the ASSENT-3 trial has multiple recurrent events. The core function in these two packages generates two times of survival and censoring times $T_i$, and $C_i$; the event occurs if $T_i \le C_i$, and for multiple events, it generates multiple survival times for each subject which represents a counting process [15, 17]. For recurrent events, the core function generates the numbers of episodes from a separate and independent distribution, then follows a similar approach as above. ASSENT-3 trial follows each subject for 30 days unless the subject dies, so none of the subjects are censored, also it has multiple recurrent events; therefore, we used our specific simulation in this study.

In order to generate data sets based on the ASSENT-3 trial results, we consider a subject at index (time 0) at risk for four types of events. Following each non-fatal event, the clock restarts only stopping if the subject dies or reaches a combined 30 days. The events were generated following a multinomial distribution to determine the event type, and an exponential distribution to determine the time to each event. In the multinomial distribution, the rate of a ‘non-event’, or event-free survival, is one minus summation of other rates. The consideration of a ‘non-event’ allows us to consider a mean time of ‘non-event’ rather than censoring time $C_i$ similar to Morina et al. [15] and Brilleman et al. [16]; we estimate a mean time of a non-event. For mean time-to-events, we assumed

$$\begin{aligned} \mu _{REMI}+\mu _{CHF}+\mu _{Shock}+\mu _{Death}+\mu _{No-event}=30-\mu _{time \ after \ death}. \end{aligned}$$

The bounds on ‘non-event’ time can be developed considering the extreme cases of time to death events. Assuming a death rate of 3%, and that all the deaths occur at the last day of followup period (i.e., day 30), $\mu _{time \ after \ death}=0$, and the expected time of no-event is $\mu _{No-event}=16.8$. At the other extreme, if we assume that all the death events occurred at the beginning of the trial period, then $\mu _{time \ after \ death}=0.9$, and the expected time of non-event is $\mu _{No-event}=15.9$, which gives the upper and lower bounds of the mean time of non-event,

7 $$\begin{aligned} 15.9 \leq \mu _{No-event} \leq 16.8. \end{aligned}$$

Through a recursive algorithm, given in the Appendix, with $m=1000$ repetition, each repetition with a sample of size $n=1500$, we observed that if we start with $\mu _{No-event}=1$, the value of $\mu _{No-event}$ oscillates between the bounds (7), and converges to the value of 16.27 for treatment arm, Fig. B1 in the Appendix.

The event rates of ASSENT-3 trial are calculated based on 30 days of the theoretical experiment for recurrent events. Due to the possibility of of recurrent events, if we use event rates directly, the rates in the simulated experiment will increase. Therefore, we adjusted the incident rates so that we end up with event rates similar to the original trial. For ease of calculation, we assume that the event rates are proportional to incident rates by coefficient $\frac{1}{C}$. By simulations for *C* in the interval [1, 5], we found that $C=2.12$, and the average of event rates of 1000 simulated samples of size $n=1500$ for treatment arm are $\hat{p}_{REMI}=0.0618$, $\hat{p}_{CHF}=0.0521$, $\hat{p}_{Shock}=0.0417$, $\hat{p}_{Death}=0.0295$.

**Fig. 1:**
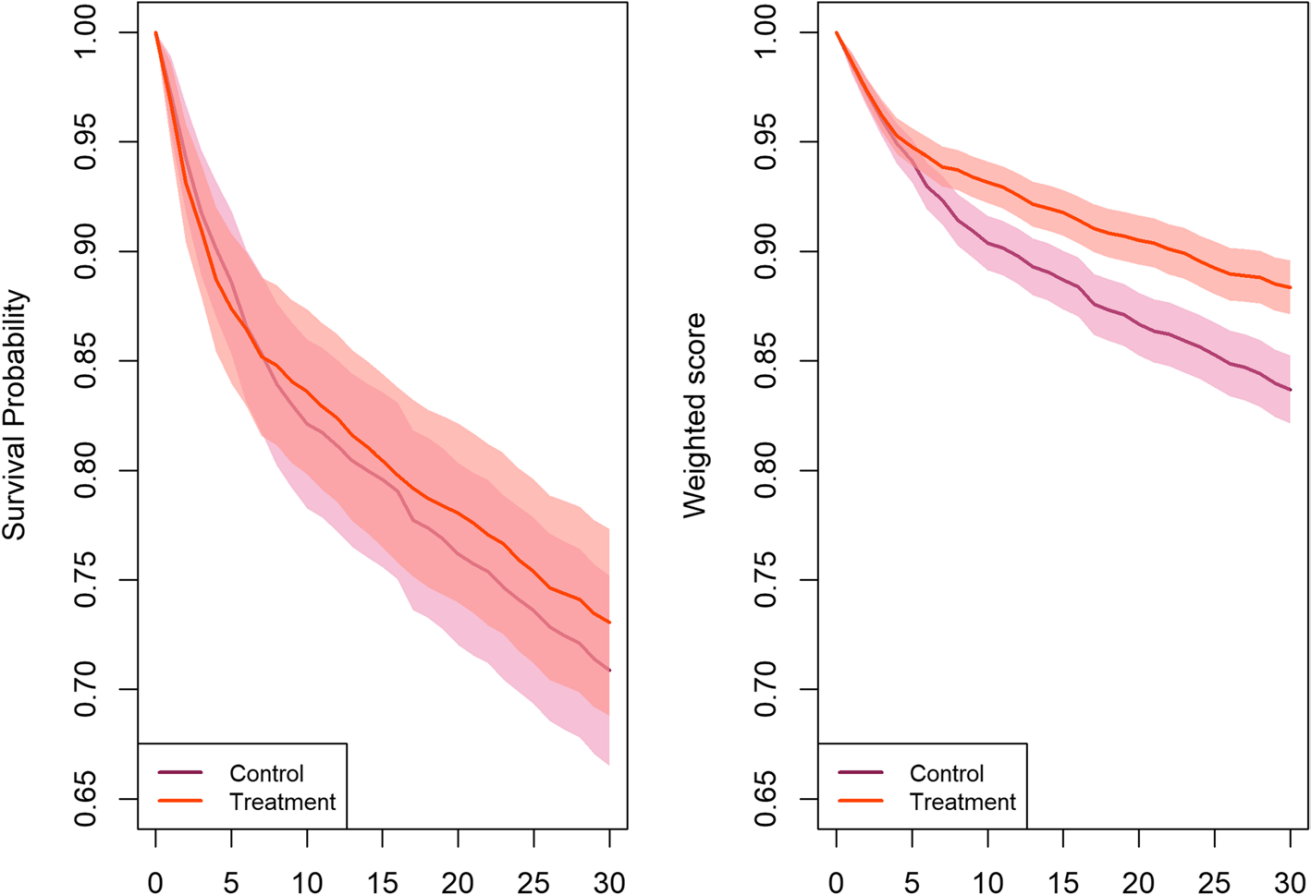
Modified K-M curves with 95% confidence intervals for a typical simulation of 1500 subjects in each arm. The left panel shows the traditional model, and the right panel shows the model with weighted composite endpoints

By using the developed simulation algorithm, we simulated samples of 1500 subjects in each arm with the given information in Table 1. We applied both the traditional composite endpoint and weighted composite endpoint analyses on this simulated example. A typical unweighted and weighted K-M are given in Fig. 1 using R package *wcep*. This figure illustrates a typical simulation result with 95% confidence interval for both analyses; and it shows the effect of considering weights of the component outcomes providing complementary insights to the time-to-first-event analysis.

## Discussion

Typical survival analysis methods are based on the assumption that there is one type of non-recurrent event (e.g., death). There is an interest in including non-fatal, clinically-relevant outcomes as they offer both the potential to increase insight into the effect of a particular treatment, but also reduce the sample size required to power a trial. However, the traditional analytic approach for composite endpoints considers only the first event, thus neglecting sequelae for subjects. Most trial design is based on a relative improvement in the composite endpoint even though given the variety of endpoints, it is unlikely to occur uniformly. Considering the qualitative, asperity and chronological components of heterogeneity is critical in evaluating the outcomes selected to determine the efficacy of a treatment.

The WCE method applies weighting to each event type to address asperity and incorporate subsequent events. The method has started seeing application in the analysis of trial data, and offers important additional insight over conventional methods [6, 18–20]. The current study addresses concerns about absence of theoretical foundation [8]. By using the events in this manner, we have shown that despite the weighting, the increase in precision of the estimate has higher power to detect differences in treatments especially in cases with heterogeneity in the efficacy on the component outcomes. The advantage is a result of the lower variance in estimates. Traditional survival analysis of composite endpoints effectively assigns weights 1 for all events and 0 for no events; however, WCE allows for a range of weights between 0 and 1 (1 for death, 0 for no event), and positive values smaller than 1 for other events, thus allowing an increase in power for a given effect size, has the benefit of being proportional to actual event and adds the advantage of reducing the variance of the estimates. Also, WCE provides a higher survival estimate compared to the traditional approach of first-event. Consider a subject with the first event of shock, the traditional approach weights it by 1; however, WCE weights it by a value smaller than 1.

**Fig. 2:**
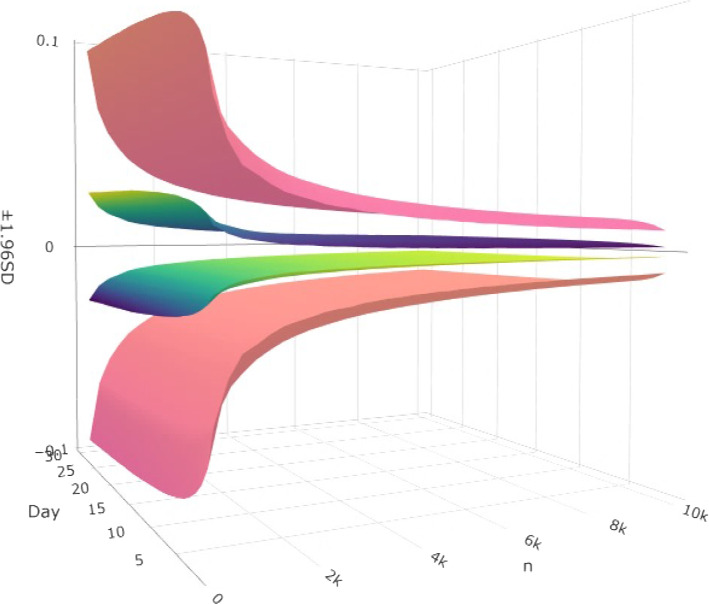
The graph shows that the predicted confidence interval get smaller by increasing sample size *n*. This figure also depicts how the WCE method provides smaller confidence interval compared to the traditional composite endpoints. Red surfaces reflect 95% margin of error of traditional composite endpoints, and greenish blue surfaces reflect the 95% margin of error for weighted composite endpoints

In order to understand the reduction in variability, consider a subject’s set of events as a uniform sample, if we put all values on 0.5, then the mean would be 0.5 and variance would be 0, whereas if we put half values on 0 and the other half on 1, the mean will be the same but the variance will be maximized. Now, if we put values between 0 and 1 the variance will be reduced. The simulated experiment demonstrated that WCE has a smaller confidence interval compared to the traditional composite endpoints, and the C.I. becomes smaller by increasing sample size *n*, Fig. 2.

Bakal et al. [12] compared the effects of the weighting against the traditional composite, through a power analysis. They noted that the weighted composite endpoint identifies differences between treatment groups more often than the traditional approach. Here, we looked at the effect of weighting on WCE variance, and compared it with the traditional composite endpoint. This study simulated samples of sizes with multiplications of 250 up to 10000, which are typical sizes for large RCTs. Similarly in applications using trials of similar size: Bakal et al. [6] with 4500 subjects, Wallentin et al. [21] with 2000 subjects, Bakal et al. [22] with 1059 subjects. In the present study, 25 samples for each size with the developed algorithm in the section 4 generated, and evaluated using both the WCE and the traditional composite endpoints. Then, the mean of 25 variances in each day of 1-30 and each approach were used as the estimate of variance for that specified approach, day, and sample size *n*. The estimated variances, 95% confidence intervals are depicted in Fig. 2, which shows that WCE provides a smaller confidence interval.

The traditional composite weights a shock similar to death, and this decrease the estimate of survival. WCE is designed to address this bias, and considers a smaller weight for shock relative death, and provides a higher survival with smaller variance compared to traditional composite. Therefore, WCE method identifies differences between treatment groups more often than the traditional approach.

In addition to the highlighted strengths of this approach, there are limitations worth noting. First, the derivation of the weights is made through a survey administered to a Delphi panel, and in this case, a cohort of experienced clinician-scientists. The overall stakeholders in ‘valuing’ these event types include subjects, healthcare providers, regulatory agencies, payors and others. Derivation and validation of global weights or stakeholder-specific weights may be useful in furthering the goal of comprehensive treatment assessment. Second, this work focusses on efficacy; there is significant clinical interest in safety endpoints, and by extension, how they relate to efficacy (i.e., net clinical benefit). Estimating benefit-risk tradeoffs remains complicated yet furtile grounds for further investigation. And finally , this approach is currently limited to time to event event types. The inclusion of longitudinal events (e.g., change in 6-minute walk distance) may deserve further investigation. With increased use, the development of a library of accepted weights would be an achievable goal. The WCE method can be difficult to apply to all trials as they may not capture all the events in a way to incorporate recurrent or multiple endpoints.

The application of WCE is restricted to RCTs with fixed followup period, say 30 days. So, all subjects have a complete followup except the subjects who die before the end of the trial period. Hence, WCE rejects right censoring or if a subject lost followup at any time during the study except death. Finally, WCE is designed for independent multiple recurrent events. Future work will explore the incorporation of censored observations and the expansion of WCE from RCTs to general survival analysis such as the semi-parametric Cox model to make inferences about explanatory covariates, and comparisons for multiple arm trials.

## Supplementary Information

**Additional file 1: Appendix.**

## Data Availability

The anonymized ASSENT-3 data and simulated data that support the findings of this study are available from the corresponding author, JB, upon reasonable request.
