# Supplementary material for: The empirical estimate of the survival and variance using a weighted composite endpoint
Source: BMC Med Res Methodol. 2023 Feb 6;23:35. doi: 10.1186/s12874-023-01857-0 (PMC9901109; doi:10.1186/s12874-023-01857-0)
Supplement: Supplementary file 1 — Additional file 1: Appendix. [file 12874_2023_1857_MOESM1_ESM.pdf]

□

APPENDIX

A PROOF OF VARIANCE

The survival function at patient level (1) looks like the product-limit estimator, and we use the same approach by delta-method approximation, similar to[23] variance for the classical life-table estimator. The idea in this approximation is given by

$$f(X) \approx f(c) + f'(c)(X - c) \tag{A1}$$

for a function $f(X)$ of a random variable X with first derivative f' and c close to $E(X)$. This implies

$$Var\,(f(X)) \approx f'(c)^2 Var\,(X)\,.$$

Define $f(X) = log(X)$; equation (1) implies

$$log\left(\hat{S}_i(t)\right) = \sum_{j:t_j \le t} log\left(1 - \omega_{ij}\right);$$

by using the result (4) and equation (A1) implies

$$\begin{aligned} log\left(\hat{S}_i(t)\right) &\approx \sum_{j:t_j \le t} \left[log(1 - W^T P_j) - \frac{1}{1 - W^T P_j} \left(\omega_{ij} - W^T P_j\right) \right] \\ &\approx C - \sum_{j:t_j \le t} \frac{1}{1 - W^T P_j} \left(\omega_{ij} - W^T P_j\right). \end{aligned} \tag{A2}$$

In Greenwood formulation $d_1, \cdots, d_{j-1}$ affects the risk set counts n_j . Then, it is proved that the variance of the sum is the sum of the variances. However, in our case $\frac{d_j}{n_j}$ replaced by ω_{ij}, and $\hat{S}_i(t)$ is survival of only one patient, not all patients. We assumed multiple recurrent events in a patient are sequentially multiplicative and independent. Note that the weight w_{SHK} dose not change after a shock, or REMI. Therefore,

$$Var\left(log\left(\hat{S}_i(t)\right)\right) \approx \sum_{j:t_j \le t} \frac{1}{(1 - W^T P_j)^2} W^T \left[diag(P_j) - P_j P_j^T\right] W;$$

and a second application of (A1) for $f(X) = e^X$ implies that

$$Var\left(\hat{S}_i(t)\right) \approx \hat{S}_i^2(t) \sum_{j:t_j \le t} \frac{1}{(1 - W^T P_j)^2} W^T \left[diag(P_j) - P_j P_j^T\right] W.$$

Finally by applying (2) and independent patients

$$Var\left(\hat{S}(t)\right) \approx \frac{1}{n^2} \sum_{i=1}^{n} \hat{S}_i^2(t) \sum_{j:t_j \le t} \frac{1}{(1 - W^T P_j)^2} W^T \left[diag(P_j) - P_j P_j^T\right] W.$$

B TOY EXAMPLE

Patient ID	Event type	Event time
1	DTH	3
2	No event	30
3	CHF	9
4	No event	30
5	No event	30
6	No event	30
7	No event	30
8	No event	30
9	SHK	7
10	No event	30
11	No event	30
12	No event	30
13	SHK	4
14	DTH	16
15	No event	30
16	CHF	16
17	DTH	8
18	CHF	1
18	SHK	5
18	SHK	10
18	DTH	15
19	SHK	2
20	CHF	5
21	REMI	1
22	No event	30
23	No event	30
24	No event	30
25	No event	30

TABLE B1 Toy example with four events of CHF, REMI; Shock, and Death, 25 patients and 30 days follow up.

Patient ID	Time t_j												
	0	1	2	3	4	5	7	8	9	10	15	16	30
1	1	1	1	0	0	0	0	0	0	0	0	0	0
2	1	1	1	1	1	1	1	1	1	1	1	1	1
3	1	1	1	1	1	1	1	1	0.7	0.7	0.7	0.7	0.7
4	1	1	1	1	1	1	1	1	1	1	1	1	1
5	1	1	1	1	1	1	1	1	1	1	1	1	1
6	1	1	1	1	1	1	1	1	1	1	1	1	1
7	1	1	1	1	1	1	1	1	1	1	1	1	1
8	1	1	1	1	1	1	1	1	1	1	1	1	1
9	1	1	1	1	1	1	0.5	0.5	0.5	0.5	0.5	0.5	0.5
10	1	1	1	1	1	1	1	1	1	1	1	1	1
11	1	1	1	1	1	1	1	1	1	1	1	1	1
12	1	1	1	1	1	1	1	1	1	1	1	1	1
13	1	1	1	1	0.5	0.5	0.5	0.5	0.5	0.5	0.5	0.5	0.5
14	1	1	1	1	1	1	1	1	1	1	1	0	0
15	1	1	1	1	1	1	1	1	1	1	1	1	1
16	1	1	1	1	1	1	1	1	1	1	1	0.7	0.7
17	1	1	1	1	1	1	1	0	0	0	0	0	0
18	1	0.7	0.7	0.7	0.7	0.35	0.35	0.35	0.35	0.175	0	0	0
19	1	1	0.5	0.5	0.5	0.5	0.5	0.5	0.5	0.5	0.5	0.5	0.5
20	1	1	1	1	1	0.5	0.5	0.5	0.5	0.5	0.5	0.5	0.5
21	1	0.8	0.8	0.8	0.8	0.8	0.8	0.8	0.8	0.8	0.8	0.8	0.8
22	1	1	1	1	1	1	1	1	1	1	1	1	1
23	1	1	1	1	1	1	1	1	1	1	1	1	1
24	1	1	1	1	1	1	1	1	1	1	1	1	1
25	1	1	1	1	1	1	1	1	1	1	1	1	1
n_{j+1}	25.0	24.5	24.0	23.0	22.5	21.65	21.15	20.15	19.85	19.68	19.5	18.2	18.2
d_j	0.00	0.50	0.50	1.00	0.50	0.85	0.50	1.00	0.30	0.18	0.18	1.30	0.00
$(1 - d_j/n_j)$	1.00	0.98	0.98	0.96	0.98	0.96	0.98	0.95	0.99	0.99	0.99	0.93	1.00
$\hat{S}(t_{j-1})(1 - d_j/n_j)$	1.00	0.98	0.96	0.92	0.90	0.87	0.85	0.81	0.79	0.79	0.78	0.73	0.73
$\frac{1}{n}\sum_{i=1}^{n}\hat{S}_i(t_j)$	1.00	0.98	0.96	0.92	0.90	0.87	0.85	0.81	0.79	0.79	0.78	0.73	0.73

TABLE B2 Life table of the toy example with calculation of survival functions based on Bakal's method and its equality with average.

C R-PACKAGE FOR WEIGHTED COMPOSITE ENDPOINTS

R-package *wcep* containing code to perform the described method in the article, and the complete data set of the toy example, used in the article. It is available from the Comprehensive R Archive Network (CRAN) at *https://CRAN.R-project.org/package=wcep*.

D R CODE FOR RECURSIVE ALGORITHM

This code is used to simulate 1000 samples of size 1500 for Figure B1 . The generating functions are provided before the recursive algorithm.

```
#function for generating multinomial events
gen<-function(p){
which(rmultinom(1, size = 1, prob = p)==1)}

#Generating events for a patient during the experiment 30 days
onesample<-function(){
time_event<-NULL
repeat{
m<- ifelse(is.null(dim(time_event)), 1, prod(p[time_event[,1]]))
px<-p
px[1:4]<-m*p[1:4]
px[5]<-1-sum(px[1:4])
event<-gen(px)
repeat{
```

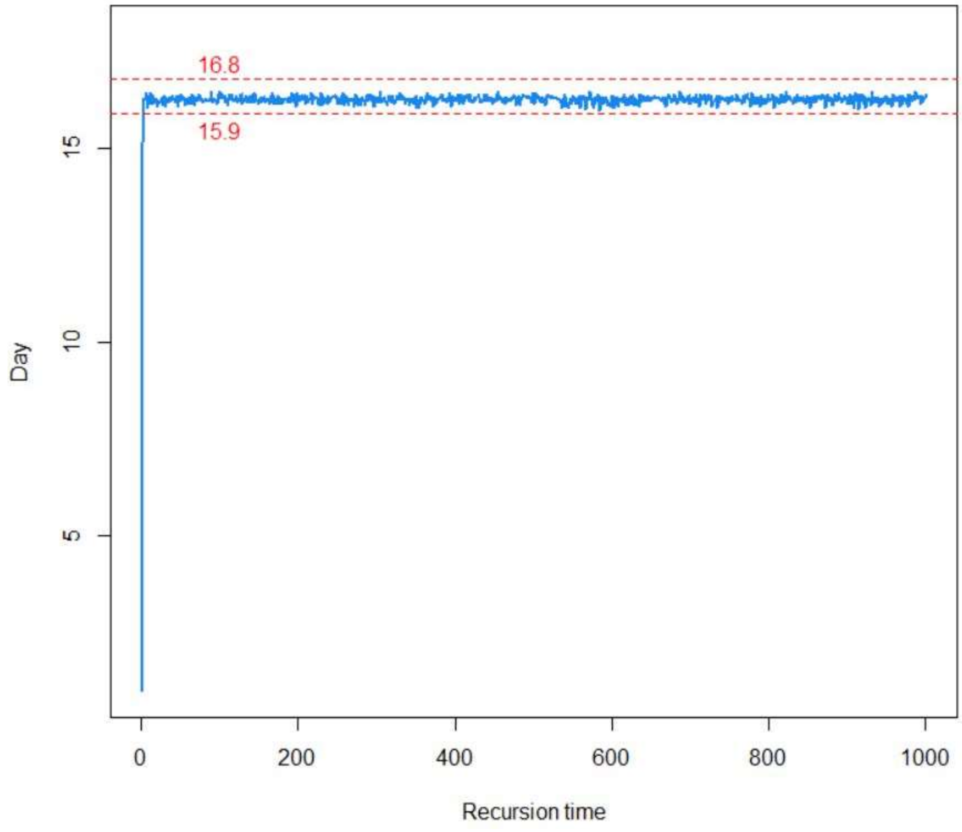

FIGURE B1 The mean time for no-event $\mu_{No-event}$ converges fast from 1 to 16.27 days for the treatment arm and moves between two extreme bounds.

```
time<-ceiling(rexp(1,1/mn[event]))
if(time!=0) {break}
}
time_event<-rbind(time_event,c(event,time))
#Death is event 4 and no-event is 5
if(apply(time_event,2,sum)[2]>=exp_time | event==4){break}
}
cs<-cumsum(time_event[,2])
time_event[,2]<-cs
if(dim(time_event)[1]==1 && time_event[1,1]==5 &&
time_event[1,2]>exp_time) {time_event[1,2]=exp_time}
if(dim(time_event)[1]==1 && time_event[1,1]!=5 &&
time_event[1,2]>exp_time) {time_event[1,2]=exp_time;
time_event[1,1]=5}
if(length(which(time_event[,2] > exp_time))!=0)
{TE<-matrix(time_event[-which(time_event[,2] > exp_time),],
ncol=2)} else {TE<-matrix(time_event,ncol=2)}
if((length(unique(TE[,1]))==1) && (unique(TE[,1])==5))
{TE<-matrix(TE[which(TE[,2]==max(TE[,2])),],ncol=2)}
else {TE<-matrix(TE[which(TE[,1]!=5),],ncol=2)}
TE<-data.frame(Event=TE[,1],TIME=TE[,2])
if(dim(TE)[1]==1 && TE[1,1]==5 && TE[1,2]<exp_time)
```

```
{TE[1,2]<-exp_time}
TE
}

#function of generating experiment of a sample of size n patients
GS <- function(n){
samp<-data.frame(ID=0,Event=0,TIME=0)
for (i in 1:n) {
ID<-i
x<-cbind(ID,onesample())
samp<-rbind(samp,x)
}
samp[-1,]
}

#Recursive algorithm
exp_time<-30
#Event rates
p<-c(0.06,0.05,0.04,0.03,0.82)
#original probability
C<-2.12
p[1:4]<-p[1:4]/C
p[5]<-1-sum(p[1:4])
#mean time-to-event
mn<-c(3,2.4,2.3,5.5,1)
#sample size
n=1500
mu1<-1
prop<-NULL
pr<-NULL
for (i in 1:1000) {
samp<-GS(n)
samp<-samp
Edeath<-samp[which(samp[,2]==4),]
mu=(n*30-sum(30-Edeath[,3]))/n-sum(mn[-5])
mn[5]<-mu
mu1<-c(mu1,mu)
prop<-rbind(prop,table(samp[,2]))
pr<-rbind(pr, c(length(unique(samp[which(samp[,2]==1),1])),
length(unique(samp[which(samp[,2]==2),1])),
length(unique(samp[which(samp[,2]==3),1]))))
}
apply(prop,2,mean)/1500
apply(prop[10:100,4:5],2,mean)))

plot(mu1,type="l", lwd=2, xlab="Recursion time", ylab="Day",
col="dodgerblue2", ylim=c(1,18))
abline(h=15.9, lty=2, col='red')
abline(h=16.8, lty=2, col='red')
text(100,15.5,"15.9", col='red')
text(100,17.2,"16.8", col='red')
```

References

1. Oulhaj A, El Ghouch A, Holman RR. Testing for qualitative heterogeneity: An application to composite endpoints in survival analysis. *Statistical methods in medical research* 2019; 28(1): 151–169.

2. Shaikh A, Ochani RK, Khan MS, et al. Contribution of individual components to composite end points in contemporary cardiovascular randomized controlled trials. *American heart journal* 2020; 230: 71–81.

3. Mao L, Kim K. Statistical models for composite endpoints of death and non-fatal events: a review. *Statistics in Biopharmaceutical Research* 2021; 13(3): 1–26.

4. Andersen PK, Gill RD. Cox's regression model for counting processes: a large sample study. *The annals of statistics* 1982: 1100–1120.

5. Pocock SJ, Ariti CA, Collier TJ, Wang D. The win ratio: a new approach to the analysis of composite endpoints in clinical trials based on clinical priorities. *European heart journal* 2012; 33(2): 176–182.

6. Bakal JA, Roe MT, Ohman EM, et al. Applying novel methods to assess clinical outcomes: insights from the TRILOGY ACS trial. *European heart journal* 2015b; 36(6): 385–392.

7. Armstrong PW, Westerhout CM, Werf V. dF, et al. Refining clinical trial composite outcomes: An application to the Assessment of the Safety and Efficacy of a New Thrombolytic–3 (ASSENT-3) trial. *American heart journal* 2011; 161(5): 848–854.

8. Ozga AK, Rauch G. Weighted composite time to event endpoints with recurrent events: comparison of three analytical approaches. *BMC Medical Research Methodology* 2022; 22(1): 1–14.

9. Armstrong PW, Westerhout CM. The power of more than one. *Circulation* 2013; 127(6): 665–667.

10. Stafinski T, Menon D, Nardelli A, et al. Incorporating patient preferences into clinical trial design: results of the opinions of patients on treatment implications of new studies (OPTIONS) project. *American heart journal* 2015; 169(1): 122–131.

11. Sinnaeve PR, Alexander JH, Bogaerts K, et al. Efficacy of tenecteplase in combination with enoxaparin, abciximab, or unfractionated heparin: one-year follow-up results of the Assessment of the Safety of a New Thrombolytic-3 (ASSENT-3) randomized trial in acute myocardial infarction. *American heart journal* 2004; 147(6): 993–998.

12. Bakal JA, Westerhout CM, Armstrong PW. Impact of weighted composite compared to traditional composite endpoints for the design of randomized controlled trials. *Statistical methods in medical research* 2015a; 24(6): 980–988.

13. Investigators of A. Efficacy and safety of tenecteplase in combination with enoxaparin, abciximab, or unfractionated heparin: the ASSENT-3 randomised trial in acute myocardial infarction. *The Lancet* 2001; 358(9282): 605–613.

14. Armstrong PW. A comparison of pharmacologic therapy with/without timely coronary intervention vs. primary percutaneous intervention early after ST-elevation myocardial infarction: the WEST (Which Early ST-elevation myocardial infarction Therapy) study. *European heart journal* 2006; 27(13): 1530–1538.

15. Moriña D, Navarro A, others . The R package survsim for the simulation of simple and complex survival data. *Journal of Statistical Software* 2014; 59(2): 1–20.

16. Brilleman SL, Wolfe R, Moreno-Betancur M, Crowther MJ. Simulating Survival Data Using the simsurv R Package. *Journal of Statistical Software* 2021; 97(1): 1–27.

17. Aalen O. Nonparametric inference for a family of counting processes. *The Annals of Statistics* 1978: 701–726.

18. Ribeiro MH, Campos CM, Padilla L, et al. Risk Burden of Coronary Perforation in Chronic Total Occlusion Recanalization: Latin American CTO Registry Analysis. *Journal of the American Heart Association* 2022; 11(11): e024815.

19. Hara H, Klaveren vD, Takahashi K, et al. Comparative methodological assessment of the randomized GLOBAL LEADERS trial using total ischemic and bleeding events. *Circulation: Cardiovascular Quality and Outcomes* 2020; 13(8): e006660.

20. Capodanno D, Gargiulo G, Buccheri S, et al. Computing methods for composite clinical endpoints in unprotected left main coronary artery revascularization: a post hoc analysis of the DELTA registry. *Cardiovascular Interventions* 2016; 9(22): 2280–2288.

21. Wallentin L, Goldstein P, Armstrong P, et al. Efficacy and safety of tenecteplase in combination with the low-molecular-weight heparin enoxaparin or unfractionated heparin in the prehospital setting: the Assessment of the Safety and Efficacy of a New Thrombolytic Regimen (ASSENT)-3 PLUS randomized trial in acute myocardial infarction. *Circulation* 2003; 108(2): 135–142.

22. Bakal JA, Westerhout CM, Cantor WJ, et al. Evaluation of early percutaneous coronary intervention vs. standard therapy after fibrinolysis for ST-segment elevation myocardial infarction: contribution of weighting the composite endpoint. *European heart journal* 2013; 34(12): 903–908.

23. Greenwood M. The natural duration of cancer (report on public health and medical subjects no 33). *London: Stationery Office* 1926.
